# Pertussis outbreak in southern Ethiopia: challenges of detection, management, and response

**DOI:** 10.1186/s12889-020-09303-2

**Published:** 2020-08-11

**Authors:** Aychiluhim D. Mitiku, Mesele D. Argaw, Binyam F. Desta, Zergu T. Tsegaye, Afework A. Atsa, Bekele B. Tefera, Ephrem Teferi, Deirdre Rogers, Ismael A. Beshir, Asrat G. Alemu, Desta A. Ayesa, Derebe T. Abate, Agegnehu G. Sendeku, Rudzani Muloiwa

**Affiliations:** 1 USAID Transform: Primary Health Care project, JSI Training & Research Institute, Inc. in Ethiopia, P.O. Box 1392, code 1110 Addis Ababa, Ethiopia; 2 Daro Malo Woreda Health Office, Dara Malo, Ethiopia; 3 USAID Transform: Primary Health Care project, Pathfinder International, Addis Ababa, Ethiopia; 4 JSI Research & Training Institute, Inc., Boston, USA; 5 Department of Paediatrics & Child Health, Groote Schuur Hospital & University of Cape Town, Cape Town, South Africa

**Keywords:** Pertussis, Outbreak, Investigation, Dara Malo, Southern Ethiopia

## Abstract

**Background:**

Despite the availability of effective vaccines, pertussis remains endemic with high fatality rates in low and middle-income countries (LMIC). This study aims to describe an outbreak of pertussis in a health district of Ethiopia. The study highlights the challenges faced by the health system in identifying pertussis cases and appropriately responding to the outbreak at the district level.

**Methods:**

A descriptive cross-sectional study was conducted using data sourced from the District Public Health Emergency and Management (PHEM) surveillance service and outbreak management field reports. Stratified attack rates and fatality rates for pertussis are described. Systemic problems leading to the outbreak are explored and narrated. A modified CDC pertussis case definition was employed with a polymerase chain reaction used to confirm cases.

**Results:**

From September 2018 to January 2019, 1840 suspected, probable, and confirmed pertussis cases and six deaths were identified. Pertussis cases ranged from 1 month to 51 years in age. An outbreak occurred in 14 out of the 24 villages of Dara Malo district. The overall attack rate was 1708 per 100,000 population with a fatality rate of 3.3 per 1000 pertussis cases. The highest attack rate of 12,689/100,000 was seen in infants. Among confirmed, probable and suspected pertussis cases, only 41.1% had completed the three-dose pertussis vaccine’s primary schedule. The household survey revealed a population coverage of 73.4 and 40.8% for Pentavalent vaccine dose one and three respectively. Investigations suggested the existence of a poor cold chain management system in the study area.

**Conclusions:**

There is an urgent need to build capacity to strengthen routine vaccination services and improve the maintenance of the vaccine cold chain. Other LMICs are urged to take lessons learned from this outbreak to strengthen their own vaccination programs and capacitate health workers to manage local outbreaks.

## Background

Pertussis is an extremely infectious vaccine-preventable disease caused by the gram-negative coccobacillus, *Bordetella pertussis*, and less commonly by *Bordetella parapertussis* [1]. Pertussis remains endemic and has reemerged as a public health problem in many countries despite decades of high vaccination coverage in infants [2]. Pertussis is widely distributed in many countries throughout the world. Globally, around 24.1 million pertussis cases and 160,700 deaths from pertussis were reported in children younger than five years in 2014. The African region contributed the largest proportion with 7.8 million (33%) cases and 92,500 (58%) deaths. Infants and young children have remained most susceptible to pertussis-related morbidity and mortality. Around 5.1 million (21%) estimated pertussis cases and 85,900 (53%) estimated deaths were in infants younger than one year [3, 4].

In children, pertussis presents classically with paroxysms of cough ending with the characteristic whoop and post-tussive vomiting. However, in young infants, pertussis can initially present as apneic or cyanotic episodes prior to the development of cough [5]. The mode of transmission is person-to-person via aerosolized respiratory droplets or by direct contact with respiratory secretions. In its early catarrhal stage, pertussis is highly contagious, with a secondary attack rate of up to 90% among non-immune household contacts. Untreated patients may transmit infection for up to three weeks or more following the onset of typical coughing attacks, although communicability diminishes rapidly after the catarrhal stage [6].

The most common complication and the cause of most pertussis-related death is secondary bacterial pneumonia. Young infants are at the highest risk of acquiring pertussis-associated complications [7]. Adolescents and adults are an important reservoir for *B. pertussis* and are often the source of infection for children [8, 9].

Vaccination is the best way to prevent pertussis in all age groups [10]. Evidence suggests that high coverage with highly efficacious vaccines leads to high levels of protection in children under five years of age. In contrast, even minor reductions in overall coverage can lead to an increase in cases [11]. Completion of a three-dose pertussis-containing vaccine schedule prevents 80% of cases and 95% of deaths. Incomplete immunization has been shown to prevent severe morbidity with one dose and two doses decreasing mortality by 50 and 80%, respectively [12, 13].

In Ethiopia, a pertussis whole-cell vaccine (wP) has been in use since 1980 [14]. From 2007 this has been in a pentavalent formulation that in addition to pertussis whole-cell vaccine, also contains Diphtheria, Tetanus, Hepatitis B, and *Haemophilus influenza* type B (DTP-HepB1-Hib1) vaccines. The pentavalent vaccine is given in three doses scheduled at 6, 10, and 14 weeks of birth [14, 15].

Based on World Health Organization (WHO) estimates, the Ethiopian national coverage of one dose of pertussis-containing vaccines in 2018 was 85%, while that of three doses was 72%, which is less than the average coverage for Sub Saharan countries [10]. This immunization coverage differs greatly between districts and regions of Ethiopia with coverage of 3 doses of pentavalent vaccine ranging from 20.1% in Afar to 81.4% in Tigray. The difference in vaccine coverage is partly due to geographical inaccessibility and other social determinants [16]. However, according to the routine health management information system (RHMIS) report, the Dara Malo district health officials reported a higher vaccine coverage with the third pentavalent dose coverage of 103.0% in 2016/2017 and 94.8% in 2017/2018. The pentavalent vaccine dropout rates were 3.0 and 3.4% respectively over the same period [17].

Diagnosis of pertussis, which is largely based on the clinical picture, may be modified by age, history of previous immunization or infection, antibiotic exposure, and concurrent infection with other pathogens [18]. According to the WHO vaccine-preventable diseases 2019 global summary report, there were no reported pertussis cases from 2000 to 2017 in Ethiopia. In 2018, however, there were 2423 cases of pertussis reported in Ethiopia [15]. WHO data is further corroborated by independent data sources that indicated the occurrence of cases of pertussis in Ethiopia [19, 20].

Uncoordinated and poor epidemic response mechanism is a challenge in containing epidemics and in reducing the resultant morbidity, mortality, and economic loss in different countries. This study aims to describe an outbreak of pertussis in the Dara Malo district of Gamo Administrative Zone, Southern Ethiopia, and to investigate possible factors that may have led to the outbreak. In addition, the study explores and highlights the challenges faced by a low and middle-income country’s (LMIC) health care system in case identification and management in response to a pertussis outbreak.

## Methods

### Study area and population

Dara Malo is one of 160 districts administered under Southern Nations, Nationalities and Peoples Region (SNNPR), Ethiopia. The district has 24 administrative structures consisting of one urban and 23 rural villages [17]. Based on the 2007 Ethiopian national census, the projected population of Dara Malo district in 2018 was 107,715 with 16,814 under five and 3436 under one year of age [17].

### Study design and sampling methods

To investigate the outbreak a cross-sectional descriptive study design was employed. In addition, based on WHO recommendations for evaluation of vaccination coverage a multistage cluster random sampling method was used [21].

All surveillance records collected from 1st September 2018 to 9th January 2019 were reviewed to identify all documented suspected, probable, and confirmed pertussis cases during the period. Health records were reviewed for each of these cases.

The sample size (*n)* for household survey was calculated using single population formula:

$$n=\frac{{Z^2}_{\raisebox{1ex}{$\alpha $}\!\left/ \!\raisebox{-1ex}{$2$}\right.}p\left(1-p\right)}{d^2}$$ was employed [22].

Where *ni* is sample size, *P* is the proportion of third dose pentavalent vaccination coverage [23], and *d* is the margin of error.

The following assumption was used: since P is 0.53% (*p* = 0.53, q = 0.47), allowing 5% for expected margin of error (*d*) with 95% confidence level (Z _α/2_ = 1.96), considering a design effect of 1.5 and 10% for non-response rate the required sample size *n* is **631**. After allocating the required sample size for each village using probability populational to size sampling method, primary data were collected from households selected using a systematic random sampling technique.

The primary sampling units were Women Development Army (WDA) members or Health Development Army (HAD) members. The WDA strategy was established in 2018 to enhance community health service quality and equity in Ethiopia. WDA is a network of one to five households represented by their team leaders. Based on geographic location of neighbourhoods, each WDA network is comprised of 25 to 30 households [24]. A list of 720 development teams was organized by the Daro Malo District Health Office [17]. For the first stage, 144 development teams were selected using random table numbers. For the second stage, eighteen to twenty four households in six segments (development teams) of villages were selected using a lottery system by local community leaders [25]. Mothers with children aged 5–23 months of age were interviewed at their homes by trained data collectors.

### Data collection

In the pertussis case review component of the study, data were sourced from the Public Health Emergency and Management (PHEM) database that includes surveillance data on pertussis. Data extracted included information on age, sex, geographical location, immunization status, dates of onset and health facility visits, treatment received, and outcomes of reported pertussis cases. The study reviews available data to explore the cause of the outbreak in terms of a functional cold chain, and vaccines supply systems at the targeted health facilities and district health office.

In addition, to verify the reported immunization coverage, a household survey was conducted. Mothers with children aged 5–23 months of age were interviewed face to face at their homes by trained data collectors. The household survey questionnaires gathered information on sociodemographic characteristics of mothers (care-takers) and the youngest child. Moreover, childhood immunization date and dose related information was obtained from individual health cards as a preferred source. However, in the absence of childhood immunization cards, the mothers’ reports (verbal recall) were taken.

The data were collected by four trained health professionals, each with a master’s degree in public health (MPH) and prior experience in reviewing documents. In addition, project advisors and program officers from Transform: Primary Health Care (TPHC), a USAID program, oversaw the quality of collected data.

To ensure quality of data collection, training was provided to the data collectors and their supervisors. At the end of every data collection day, a meeting was held between the data collectors and a supervisor to discuss practical problems and issues of major concern. The investigators rechecked the completeness of the collected data on a daily basis.

#### Case definition

To confirm pertussis, nasopharyngeal samples were taken from suspected cases by experienced laboratory professionals from the Ethiopian Public Health Institutes and tested using polymerase chain reaction (PCR) for *Bordetella pertussis* and *Bordetella parapertussis* (RealStar® Bordetella PCR Kit 1.0. Altona Diagnostics). Suspected cases with paroxysmal cough illness for more than 2 weeks were assessed for possible epidemiological links to cases confirmed by a positive PCR for *Bordetella pertussis*. Pertussis cases reported to the district health office PHEM department were classified by the outbreak investigation team as laboratory-confirmed, probable, or suspected pertussis using modified Center for Diseases Prevention and Control (CDC) criteria [26].

Confirmed: A confirmed pertussis case is defined as acute cough illness of any duration with a positive PCR for *B. pertussis*, or a case that meets the clinical case definition and is epidemiologically linked directly to a lab-confirmed case [23, 24]. In this study, the confirmed pertussis case is an acute cough illness of any duration with a positive PCR for *B. pertussis*.

Probable: A probable case is defined as a cough lasting ≥2 weeks AND paroxysms of coughing, inspiratory “whoop” or post-tussive vomiting AND No laboratory confirmation AND No epidemiologic linkage to a lab-confirmed case. In this study, probable pertussis case is a case Cough lasting ≥2 weeks AND Paroxysms of coughing, inspiratory “whoop” or post-tussive vomiting, AND No laboratory confirmation [10, 26].

Suspected: A suspected case is defined as a non-improving cough of 14 days or more or cough of any duration with paroxysms, or cough of any duration with a whoop.

##### An epidemic of pertussis

According to the CDC pertussis epidemic is defined as a situation when two or more cases clustered in time.

- ≥2 PCR confirmed cases clustered in time (within 42 days of each other) and space (e.g. in one building) where transmission is suspected to have occurred in that setting (e.g. nosocomial transmission in a hospital) OR
- 1 PCR confirmed case AND 1 epi-linked case with cough illness lasting ≥2 weeks with one of the following: paroxysms of coughing, inspiratory whoop, or post-tussive vomiting [10, 26].

#### Data management and analysis

The data were collected in a spreadsheet, cleaned, summarized, and analysed using Microsoft Excel® and Statistical Packaged for Social Sciences Research (SPSS IBM V 20) [27].

Proportions of cases have been presented as percentages. All continuous data were summarized using medians and ranges. The frequency of cases was reported as a case attack per 100,000 population using the 2018 population for Dara Malo as the denominator, while deaths were reported as fatality rates per 1000 cases. Epidemic curves were depicted using the date of onset of paroxysmal cough and date cases were identified by health workers. This analysis shows the propagated nature of the outbreak. Data were reported stratified by age groups and geographical locations and described in terms of time, place, and person.

The household survey data were used to determine population immunization coverage and to verify the accuracy of routine EPI reports. The first and third doses of pentavalent vaccination were recounted from the register, tally sheet, and compared with the caretakers’ report. To characterize the report consistency, national recommended categories were used i.e. consistent if the ratio reported was ≥90.0 and < 110.0%; overreported if it was < 90.0%; and underreported if the ratio was ≥110.0% [28].

The result of the quantitative analysis is presented in frequency tables and graphs, while the findings of filed reports and observations were thematically analysed to explain possible factors for the reported pertussis outbreak.

In addition, using ArcGIS 10.8 a spatial analysis was made to show most affected villages using attack rates and case-fatality rates.

#### Ethical clearance

Ethical clearance was obtained from both JSI Research & Training Institute, Inc., and the SNNPR Health Bureau, Ethiopia, Institute Review Boards (IRBs). Permission to conduct the study was obtained from facility managers.

## Results

### Rumour investigation

On 31st August 2018, a community member reported the death of a 72 months old girl with a history of prolonged paroxysmal cough, post-tussive vomiting, and respiratory whoops. Three more children had recently died in the same village, when a similar illness had been observed to affect almost all children in the village during August 2018.

On September 1st, 2018, the Dara Malo district Health Office revitalized the rapid response multi-sectoral committee and reviewed its Risk and Emergency Management Plan. On the same date, the district health office requested technical, and other resources support from Gamo Zone Health Departments, SNNP regional health bureau, Ethiopian Public Health Institute (EPHI), World Health Organization, USAID’s Transform and other development partners.

The PHEM core process established and deployed an epidemic investigation team which comprised a field epidemiologist, an integrated disease surveillance response officer, and health extension workers. The team members were familiarized with the case definitions, diagnosis and treatment protocols, strengthening community-based surveillance, and enhancing community mobilization strategies.

### Verifying the pertussis outbreak

The index case, a five-year-old child with unknown vaccination status was identified from Daro Dime village on 1st September 2018. A reported pertussis suspected case presented at Daro Dime Health Center with a complaint of paroxysmal cough, post-tussive vomiting, and respiratory whoop since 2nd August 2018. The source of infection for the index cases was not documented. The case was treated with trimethoprim-sulfamethoxazole (TMP- SMZ) with a dose of trimethoprim 8 mg/kg/day, sulfamethoxazole 40 mg/kg/day in two divided doses for 14 days. The outbreak investigation team regarded this probable case as the index case of this pertussis outbreak. The district health office and Daro Dime Health Centre instituted an active case search from 4th to 13th September 2018. Over the period, 471 suspected and probable pertussis cases were identified in five kebeles. The outbreak investigation team oriented community leaders, health workers, and elders. The active case search was extended through 27th March 2019. Over the whole surveillance period, a total of 1840 suspected, probable, and confirmed pertussis cases including six deaths, were identified.

### Descriptive analysis of reported pertussis outbreak

The pertussis outbreak is described in terms of time, place, and person.

### Demographic distribution of the cases

From 1st September 2018 to 9th January 2019, there were a total of 1840 reported cases of pertussis. Of the reported cases 9 (0.5%) were confirmed (all due to *Bordetella pertussis*) while 454 (24.7%) were probable cases and the remaining1377(74.8%), suspected cases. Dara Dime village reported 838 (45.5%) of the pertussis cases. The socio-demographic characteristics of the study participants are shown in Table 1. The age of the cases ranged from 1 month to 50 years of age with a median of 36 months. Of the total cases, 230 (12.5%) were aged less than six months while 801 (42.5%) were 1 to 4 years old. Table 1.

**Table 1:** Socio-demographic characteristics of Pertussis cases in Dara Malo District, Gamo Zone, SNNPR, Ethiopia, August 2018–January 2019

Variable	Characteristics	*n* = 1840(%)	Total population
**Sex**	Male	883 (48.0)	54,935
	Female	957(52.0)	52,780
**Age groups**	≤ 5 months	230 (12.5)	1615
	6–11 months	206 (11.2)	1821
	1 - 4 years	801 (43.5)	13,378
	5–9 years	457 (24.8)	14,603
	10–14 years	136 (7.4)	13,388
	≥ 15 years	10 (0.5)	62,910
	Mean ± SD= 45.4± 42.8 months; median = 36 months; and range = 599 months		

The overall attack rate was 1708/100,000. The attack rates by age category were 12,689/100,000, 5965/100,000, and 667/100,000 reported in under one-year infants, 1 to 4 years children, and 5 or more years of children and adults, respectively. Additional file 1.

**Fig. 1:**
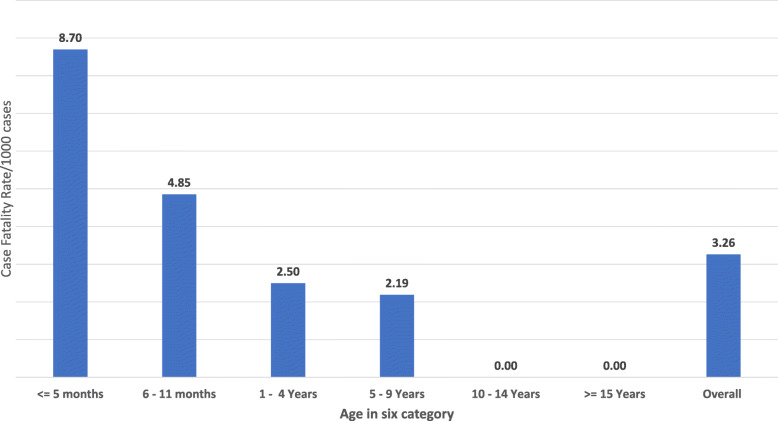
Pertussis cases fatality rate per 1000 cases by age group at Dara Malo district, Gamo Zone, SNNPR, Ethiopia, August 2018–January 2019. The chart depicts the magnitude of death by age category. The highest CFR was reported among infant age less than 5 months

A total of six deaths due to pertussis were reported to give a total case fatality rate of 3.3 deaths per 1000 cases. These include five deaths from Daro Dime and one death from Menena Aba kebele. Two deaths each were under 6 months, and between 6 to 12 months. One death each was from 1 to 4 years and 5 to 9 years. The highest case fatality rate was found among infants less than 5 months of age with a case fatality rate of 8.70/1000 cases, followed by infants aged 6 to 11 months with 4.85/1000 cases. Figure 1.

### Clinical presentation

**Fig. 2:**
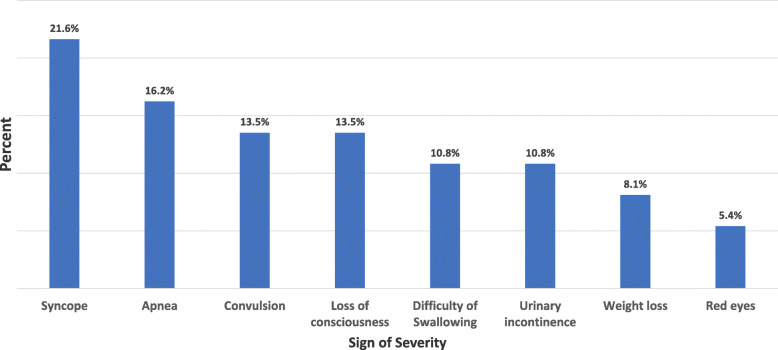
Bar chart presenting reported sings of severity among pertussis cases, cough in Dara Malo District, SNNP, August 2018 – January 2019. The figure clearly depicted the magnitude of reported signs and symptoms of severity among 43 pertussis cases. Syncope, apnoea an death the three top reported sings of severity

The most frequent presentation was paroxysmal cough which was a complaint in all 1840 (100.0%) cases. This was followed by post-tussive vomiting which was reported in 1579 (85.8%) cases. Inspiratory whoop was reported in 454 (24.7%) while 136 (7.4%) had a low-grade fever. In addition, 37(2.0%) of the cases had additional signs and symptoms indicative of severe illness. The most frequently reported signs of severity were syncope and apnea in eight (21.6%) and six (16.2%) cases, respectively. Figure 2.

### Descriptive analysis by time

**Fig. 3:**
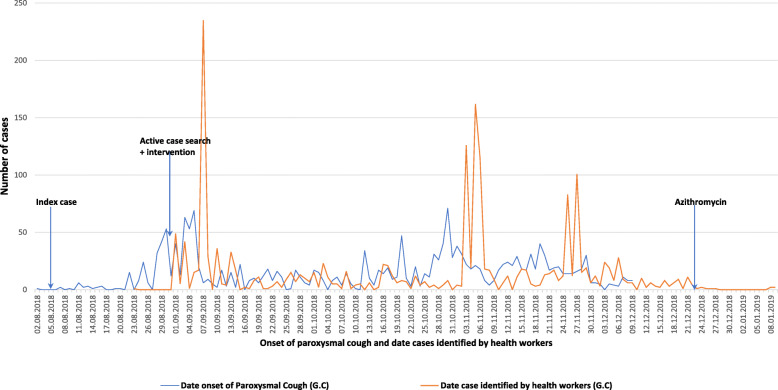
Epi Curve of Pertussis cases by date of onset of paroxysmal cough and identified by health workers in Dara Malo District, SNNP, August 2018 – January 2019. The epi-cure by date of onset of paroxysmal cough showed three peaks, which shows the propagative nature of the spread of pertussis infection. In addition, the epi-cure by date of pertussis case identified by health workers showed three peaks, which shows the long period taken to control the spread of infection

Figure 3 presents the Epi-Curve which plots the frequency of pertussis cases over time by date of onset of paroxysmal cough and date cases were identified by health workers, respectively. The epi-curve presented clearly demonstrates the classic propagated or progressive source nature of the pertussis outbreak, where three peaks are observed over time. The mean delay to seek medical care by patients or caretakers was 9.4 days, with a median of 7 days, ranging from one day to 63 days.

### Geographical distribution of the outbreak cases

**Fig. 4:**
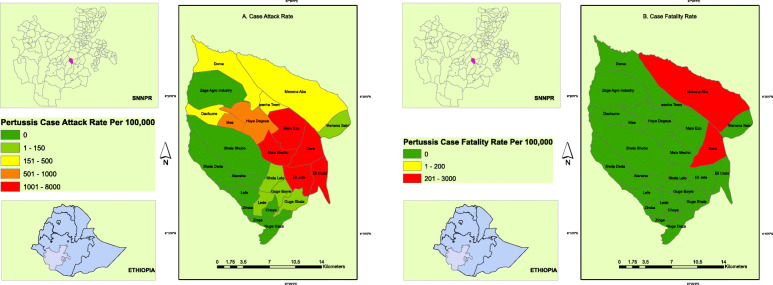
Pertussis Case Attack Rate (**a**) and Case Fatality Rate (**b**) by Village (Kebele), Dara Malo district, Gamo Zone, SNNP region, Ethiopia August 2018–January 2019. The ArcGIS map presents the location of Ethiopia, SNNPR and Dara Malo district. In addition, the Pertussis Case Attack rate and Case Fatality rates were presented with spatial analysis by village (Kebele). The map can be reproduced or used after acknowledging the authors. (Source: Shape file from Ethiopia Central Statistical Agency (CSA), 2016 at https://africaopendata.org/dataset/ethiopia-shapefiles)

The highest attack rate was reported from Dara Dime village while the highest case fatality rate was noted in *Menena Aba* village. Figure 4 depicts ArcGIS 10.8 spatial analysis pertussis case attack rates and case fatality rates by a village.

### Vaccination status

The number of cases who had received a pertussis-containing vaccine was found to be 169 (9.2%), 321 (17.4%), and 761 (41.4%) for one dose, two doses, and three doses, respectively. Of the rest of the reported pertussis cases, 232 (12.6%) had not received any vaccine dose while 357 (19.4%) had unknown immunization status. For individuals who received three doses of the pentavalent vaccine, a median of 41 (Interquartile range 19–67) months had elapsed between the last dose and the current illness.

### Management of the pertussis outbreak

There were delays in notification and lack of timely proper management of probable or suspected pertussis cases in the community. As the health workers were treating cases as pneumonia, pertussis was never suspected until the death of four children occurred due to the outbreak.

The outbreak response team lobbied community leaders and elders to mobilize the community to report cough of 2 weeks or more to nearby health posts. Information leaflets were developed and distributed to all household members using the local language, Gamo.

Individual cases were treated with antibiotics to reduce the severity and duration of symptoms, and to prevent complications. From 1st September to 22nd December 2018, 1832 (99.5%) of the identified pertussis cases were treated with amoxicillin, cotrimoxazole, or erythromycin. The outbreak response was hindered by a lack of sufficient erythromycin syrup doses for children under five years of age.

In order to reduce the spread of infection, a Mass Drug Administration (MAD) program was initiated following a facilitated two days training for 110 health workers including 90 drug distributors, 17 supervisors, and three district coordinators. The MDA was conducted from 23 to 27 December 2018 using oral azithromycin at a daily dose of 10 mg/kg under the direct supervision of health workers on the first day followed by 5 mg/kg from 2nd to 5th day. This prophylactic mass campaign targeted 69,587 residents of the 14 pertussis-affected villages. Directly observed azithromycin therapy was received by 67,236 (96.6%) people under the supervision of health care providers. Among these, 21,003 (31.2%) were under 10 years of age. The campaign required $18,325.00 USD of investment from the district health office and partners for its successful implementation.

### Prevention of spread of infection

EPI register reviews and oral responses of patients or caretakers were counted and mapped for supplementary immunization campaigns. After completing prophylactic antibiotic treatment with azithromycin, the outbreak management team organized a pentavalent vaccination campaign from the 24th to the 28th of January 2019. Three supplementary immunization campaigns were organized. Children less than two years were vaccinated, including 1697 who received their first dose, 2564 their second dose, and 3287 their third dose of pertussis-containing vaccine.

All schools were closed for the period of mass preventive prophylaxis campaigns while community members were advised to avoid participating in local markets. Community members were advised to implement strict personal hygiene including frequent hand washing and avoiding contact with pertussis suspected individuals.

Following the last reported case, the District Health Office continued its active case search for more than two months on a daily basis. The outreach investigation team continued to provide support for individual case management and mass health education for community members. It also continued to enhance the capacity of health workers on cold chain management, defaulter tracing, and use of data for decision making. After no further cases of pertussis were reported from 10th January to 27th March 2019, the investigation team was demobilized.

### Formulation of the hypothesis

The outbreak investigation team reviewed the surveillance reports filled by four health centres, fourteen health posts and six private health facilities before being organized by Dara Malo District Health Office. Based on the routine surveillance report, the completeness rate was assessed as high, with a completeness rate of 84.0% in 2016/17; 88.0% in 2017/2018; and 91.0% in 2018/2019. Poor housing conditions, reducing vaccine potency, and low vaccination coverage status of affected community were identified as a potential factors contributing to the pertussis outbreak.

### Pentavalent vaccination coverage

**Fig. 5:**
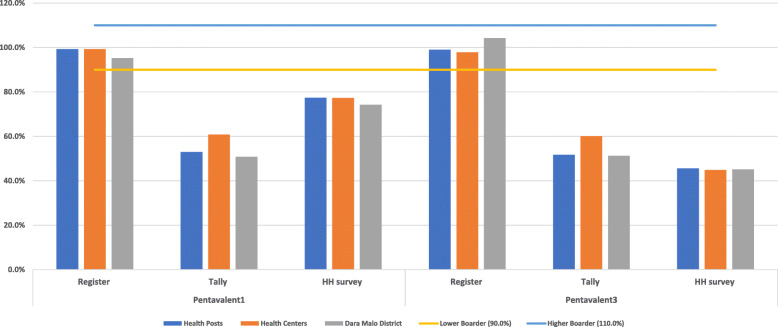
Verification of pentavalent one and three vaccination services report against household survey, July 2018 – June 2019. The bar chart shows the accuracy of routine EPI report against household survey data at health post, health centre and district level

Three hundred- and thirty-six facility months’ data were extracted. This was supplemented by survey data of 595 children aged 7–23 months, with a response rate of 94.3%. The immunization status on the health cards or caretakers’ oral reports on household surveys was compared to that sourced from register tallies. The household survey revealed a 73.4 and 40.8% vaccination coverage for pentavalent one and three doses, respectively. At the district level, EPI registers for the first and third doses of pentavalent indicated coverages of 95.2 and 104.2%, respectively. Register tallies overreported vaccination coverage by 50.8% for first pentavalent dose and by 45.1% for the third dose when compared to health card records or oral reports (Fig. 5).

### Environmental factors

The housing condition and family size per household were assessed. The average house in the affected community had 7 people and lacked air circulation. Community members were encouraged to open ventilate their homes.

### Cold chain, immunization services management

Wacha Health Centre has a high-tension electric power supply. Three out of four health centres have solar panels to obtain uninterrupted electric power for cold chain maintenance. All fourteen health centres collected vaccines for scheduled vaccination campaigns and outreach services. The investigation team did not find continuously recorded temperature monitoring tools. In addition, the thermochromic label on vials or vaccine vial monitor (VVM), revealed that the vaccines were kept at temperatures that do not preserve vaccine potency. Overall, cold chain maintenance was generally found to be poor.

## Discussion

This study confirms a pertussis outbreak that occurred between September 2018 and March 2019, in Dara Malo, a district in Southern Ethiopia. Pertussis outbreak cases occurred in 14 of 24 villages in the district and involved 1840 cases with six deaths. Pertussis is one of the under-reported major causes of morbidity and mortality among children in many low-income countries [3]. This report confirms the importance of strengthening community-based active surveillance programs and the need to enhance the capacity of community health workers in identifying pertussis suspected cases, as well as providing a prompt effective response to any outbreaks [29, 30].

The current outbreak could have been the result of poor vaccination coverage and a non-functional cold chain management system, combined with a lack of standardized national pertussis responsiveness and management guidelines. Although almost 90% of cases were older than 5 months, only 41% of them had received the three doses of a pertussis-containing vaccine as would have been appropriate for age. This report was in line with the population coverage revealed through household survey. In addition, there had been a long interval between the third pertussis vaccine dose and acquiring infection (median of 41 months); hence, the outbreak might have occurred as a result of waning vaccine acquired immunity overtime [31–33].

Though the district health officials confirmed the presence of a high rate of community-based surveillance in weekly reports on public health notifiable diseases over the duration of preceding year, the health systems did not identify the unusual health condition which occurred in Dara Malo district. This finding might have occurred due to the limited capacity of the health care system in identifying risks, developing emergency preparedness plans, as well as diagnosis and treatment of individual cases, prompt response, and management capacity.

The signs and symptoms of both uncomplicated and severe cases were consistent with either CDC or WHO pertussis case definitions for suspected pertussis cases. The cause of this outbreak was confirmed to be due to *Bordetella pertussis* on PCR. The overall pertussis case attack rate of 1708 per 100,000 inhabitants of Dara Malo district was much higher than the previously documented pertussis attack rate of 130 per 100,000 population reported in South Wollo, North Eastern Ethiopia in 2017 [19] or the 400 per 100,000 population reported in Papua New Guinea in 2012 [34]. The high attack rate documented in this investigation could be due to the case definition, which in addition to confirmed pertussis included both probable and suspected cases. Once laboratory confirmation of the outbreak was made, subsequent cases were clinically diagnosed. This practice is different from other studies, in particular those from high-income countries, in which only laboratory-confirmed and epidemiologically linked cases are considered. The highly sensitive case definition used would also have led to a relatively low case fatality rate compared to that described for pertussis in other Sub Saharan settings [35, 36].

Our report shows that there was a delay in appropriate management to control the outbreak using a nationally recommended and effective antibiotic regimen for individual cases [37]. Contact tracing was largely incomplete and the intervention did not include prophylactic antibiotic administration to susceptible community members until late in the outbreak. Failure to protect close contacts of both confirmed and probable pertussis cases might have encouraged the spread of infection over an extended period. By the time mass prophylaxis started, the outbreak was already dying out naturally. This is indicative of the lack of health system capacity to respond and manage the outbreak timeously.

The low health-seeking behaviour of the community (as indicated by the more than two months of delay in visiting the health facility recorded in some instances) was most likely due to lack of knowledge on the severity and risk of pertussis, and would have served to exacerbate the situation. As expected, the highest attack rate was in infants followed by children aged one to four years. This finding was consistent with the report of Yeung et al’s global estimates [3] and the report of the outbreak in the South Wollo area of Ethiopia [19].

The overall pertussis case fatality rate of 3.3 per 1000 pertussis cases was much lower than the 37 per 1000 pertussis cases from Mekdela [19] and the 30 per 1000 pertussis cases reported from Papua New Guinea (2012) [34]. Four of the six deaths were reported before outbreak control interventions were initiated. The early initiation of the responsive program may have averted further deaths. All cases identified during the outbreak were treated with antibiotics which consisted of amoxicillin, and cotrimoxazole, erythromycin, or azithromycin. The use of antibiotics would have reduced the possibility of severe pneumonia which can occur as a complication of pertussis infection [30].

The cause of this outbreak could be multifaceted. Even though the RHMIS reported a high rate of vaccination coverage of over 95% with three doses of a pertussis-containing vaccine in 2016/17 for Dara Malo district., a large proportion of pertussis cases were not fully vaccinated. The inconsistency between routine RHMIS and outbreak data may be due to poor data recording. Maintaining high vaccine coverage is one of the most important interventions in reducing pertussis cases and consequently averting outbreaks [11, 38, 39]. The poor vaccine vial monitoring observed during the outbreak investigation and non-functional cold chain management system at primary health care units may have resulted in poor vaccine potency even in cases that received all doses [40, 41].

### Limitations

The study was limited by a lack of resources to confirm the majority of suspected cases which would have affected the diagnostic accuracy of cases; however, in an outbreak, clinical criteria have been shown to have high sensitivity and specificity in the diagnosis of pertussis [42]. We also noted the atypical spikes in the epidemic curve that could be a result of either an artifact created by intermittent augmentation of surveillance practice or real individual clusters within the overall outbreak. In addition, this study did not capture the reason for the delayed health-seeking behaviour of caretakers and the completeness, consistency, and reliability of immunization data. As immunization records were not always available, information on vaccination status could have been affected by recall bias.

## Conclusions

There is an urgent need to build capacity to strengthen routine vaccination services, including defaulter tracing, to keep vaccine coverage at a high level to avert outbreaks. Vaccinating all children with appropriate doses for age must be supplemented with building and strengthening the capacity of health workers to timeously and properly manage outbreaks when these occur. In addition, static and outreach services, community-based surveillance activities, and cold chain maintenance need to be prioritized. A review of data quality status and capacity to respond to outbreaks at the district level must urgently be undertaken. Other LMICs, especially those in Sub-Saharan Africa, should use the lessons from this outbreak to actively review their vaccination systems and promote contextually suitable ways of outbreak detection, diagnosis, management and responses to avert similar challenges [43].

## Supplementary information

**Additional file 1: Supplementary file 1.** Pertussis cases and attack rates by village with age group in Dara Malo district, Gamo Zone, SNNPR, Ethiopia, August 2018–January 2019.

## Data Availability

The datasets was collected from the Public Health Emergency and Management (PHEM) database and used and/or analysed during the current study are available from the corresponding author on reasonable request.

## Abbreviations

AR: Attack Rate
BCC: Behavioural Change Communication
CDC: Centers for Diseases Control and Prevention
CFR: Case Fatality Rate
EPI: Expanded Programme on Immunization
FMOH: Federal Ministry of Health
HAD: Health Development Army
HEW: Health Extension Workers
HMIS: Health Management Information System
HP: Health Post
IEC: Information Education and Communication
IRB: Institution Review Board
MDA: Mass Drug Administration
MDG: Millennium Development Goal
PCR: Polymerase Chain Reaction
PHCU: Primary Health Care Unit
PHEM: Public Health Emergency and Management
SNNPR: Southern Nations, Nationalities, and Peoples’ Region
UNICEF: United Nations Children’s Fund
USAID: United States Agency for International Development
VPD: Vaccine Preventable Disease
WDA: Women Development Army
WHO: World Health Organization
