# Supplementary material for: Pertussis outbreak in southern Ethiopia: challenges of detection, management, and response
Source: BMC Public Health. 2020 Aug 11;20:1223. doi: 10.1186/s12889-020-09303-2 (PMC7422551; doi:10.1186/s12889-020-09303-2)
Supplement: Supplementary file 1 — Additional file 1: Supplementary file 1. Pertussis cases and attack rates by village with age group in Dara Malo district, Gamo Zone, SNNPR, Ethiopia, August 2018–January 2019. [file 12889_2020_9303_MOESM1_ESM.docx]

**Additional Supplementary file**

Pertussis cases and attack rates by kebele and age groups in Dara Malo district, Gamo Zone, SNNPR, Ethiopia, August 2018 – January 2019

| **Kebele /Village** | **< 1 Years population** | | | | | **1- 4 Years population** | | | | | **>= 5-year Population** | | | | | **Overall** | | | | | |
| --- | --- | --- | --- | --- | --- | --- | --- | --- | --- | --- | --- | --- | --- | --- | --- | --- | --- | --- | --- | --- | --- |
|  | **Population at risk** | **Number of pertussis cases** | **Attack rate per 1000 pop.** | **Number of deaths** | **Case fatality rate** | **Population at risk** | **Number of pertussis cases** | **Attack Rate per 1000 pop.** | **Number of deaths** | **case fatality rate** | **Population at risk** | **Number of pertussis cases** | **Attack rate per 1000 pop.** | **Number of deaths** | **Case fatality rate** | **Population at risk** | **Number of pertussis cases** | **Attack rate per 1000 pop.** | **Number of deaths** | **Case fatality rate** |  |
| Wacha town | 137 | 2 | 14.65 | 0 | 0.00 | 531 | 14 | 26.34 | 0 | 0.00 | 3611 | 1 | 0.28 | 0 | 0.00 | 4279 | 17 | 3.97 | 0 | 0.00 |  |
| Doma | 97 | 4 | 41.32 | 0 | 0.00 | 377 | 5 | 13.26 | 0 | 0.00 | 2561 | 2 | 0.78 | 0 | 0.00 | 3035 | 11 | 3.62 | 0 | 0.00 |  |
| Hoya Degeza | 87 | 7 | 80.38 | 0 | 0.00 | 339 | 9 | 26.54 | 0 | 0.00 | 2304 | 5 | 2.17 | 0 | 0.00 | 2730 | 21 | 7.69 | 0 | 0.00 |  |
| Mes (Masta) | 108 | 6 | 55.66 | 0 | 0.00 | 420 | 17 | 40.51 | 0 | 0.00 | 2852 | 4 | 1.40 | 0 | 0.00 | 3379 | 27 | 7.99 | 0 | 0.00 |  |
| Dachume (Tilale) | 101 | 1 | 9.90 | 0 | 0.00 | 393 | 6 | 15.26 | 0 | 0.00 | 2672 | 3 | 1.12 | 0 | 0.00 | 3166 | 10 | 3.16 | 0 | 0.00 |  |
| Menena Aba | 249 | 2 | 8.03 | 1 | 50.00 | 969 | 12 | 12.38 | 0 | 0.00 | 6585 | 20 | 3.04 | 0 | 0.00 | 7803 | 34 | 4.36 | 1 | 2.94 |  |
| Menena Selo | 140 | 3 | 21.39 | 0 | 0.00 | 546 | 1 | 1.83 | 0 | 0.00 | 3711 | 2 | 0.54 | 0 | 0.00 | 4397 | 6 | 1.36 | 0 | 0.00 |  |
| Zaga Agro industry | 0 | 0 | 0.00 | 0 | 0.00 | 0 | 0 | 0.00 | 0 | 0.00 | 0 | 0 | 0.00 | 0 | 0.00 | 0 | 0 | 0.00 | 0 | 0.00 |  |
| **Wacha Cluster** | **918** | **25** | 27.22 | **1** | 4.00 | **3576** | **64** | 17.90 | **0** | 0.00 | **24296** | **37** | 1.52 | **0** | 0.00 | **28790** | **126** | 4.38 | **1** | 0.79 |  |
| Malo Ezo | 172 | 58 | 336.58 | 0 | 0.00 | 671 | 127 | 189.29 | 0 | 0.00 | 4559 | 110 | 24.13 | 0 | 0.00 | 5402 | 295 | 54.61 | 0 | 0.00 |  |
| Malo Mechu | 158 | 46 | 291.96 | 0 | 0.00 | 613 | 60 | 97.81 | 0 | 0.00 | 4168 | 34 | 8.16 | 0 | 0.00 | 4939 | 140 | 28.35 | 0 | 0.00 |  |
| Dere (Dara Dime) | 334 | 176 | 526.55 | 2 | 1.14 | 1301 | 372 | 285.85 | 2 | 0.54 | 8842 | 290 | 32.80 | 1 | 0.34 | 10478 | 838 | 79.98 | 5 | 0.60 |  |
| Eli Codo | 130 | 51 | 391.47 | 0 | 0.00 | 507 | 59 | 116.32 | 0 | 0.00 | 3446 | 48 | 13.93 | 0 | 0.00 | 4084 | 158 | 38.69 | 0 | 0.00 |  |
| Eli Jele (Doze) | 214 | 78 | 365.22 | 0 | 0.00 | 832 | 112 | 134.69 | 0 | 0.00 | 5650 | 87 | 15.40 | 0 | 0.00 | 6695 | 277 | 41.37 | 0 | 0.00 |  |
| **Dara Dime Cluster** | **1008** | **409** | 405.76 | **2** | 0.49 | **3924** | **730** | 186.01 | **0** | 0.00 | **26666** | **569** | 21.34 | **1** | 0.18 | **31598** | **1708** | 54.05 | **5** | 0.29 |  |
| Shela Shubo | 179 | 0 | 0.00 | 0 | 0.00 | 698 | 0 | 0.00 | 0 | 0.00 | 4739 | 0 | 0.00 | 0 | 0.00 | 5616 | 0 | 0.00 | 0 | 0.00 |  |
| Atenshe | 53 | 0 | 0.00 | 0 | 0.00 | 205 | 0 | 0.00 | 0 | 0.00 | 1396 | 0 | 0.00 | 0 | 0.00 | 1654 | 0 | 0.00 | 0 | 0.00 |  |
| Shela Deda | 176 | 0 | 0.00 | 0 | 0.00 | 686 | 0 | 0.00 | 0 | 0.00 | 4659 | 0 | 0.00 | 0 | 0.00 | 5521 | 0 | 0.00 | 0 | 0.00 |  |
| Lefe | 134 | 0 | 0.00 | 0 | 0.00 | 521 | 0 | 0.00 | 0 | 0.00 | 3541 | 0 | 0.00 | 0 | 0.00 | 4196 | 0 | 0.00 | 0 | 0.00 |  |
| Zimbe | 61 | 0 | 0.00 | 0 | 0.00 | 238 | 0 | 0.00 | 0 | 0.00 | 1619 | 0 | 0.00 | 0 | 0.00 | 1918 | 0 | 0.00 | 0 | 0.00 |  |
| **Shela Deda Cluster** | **603** | **0** | 0.00 | **0** | 0.00 | **2348** | 0 | 0.00 | **0** | 0.00 | **15954** | **0** | 0.00 | **0** | 0.00 | **18905** | **0** | 0.00 | **0** | 0.00 |  |
| Choye | 105 | 0 | 0.00 | 0 | 0.00 | 410 | 0 | 0.00 | 0 | 0.00 | 2787 | 0 | 0.00 | 0 | 0.00 | **3303** | 0 | 0.00 | 0 | 0.00 |  |
| Lede | 133 | 0 | 0.00 | 0 | 0.00 | 518 | 0 | 0.00 | 0 | 0.00 | 3519 | 0 | 0.00 | 0 | 0.00 | **4170** | 1 | 0.24 | 0 | 0.00 |  |
| Shela Lelo | 157 | 0 | 0.00 | 0 | 0.00 | 609 | 0 | 0.00 | 0 | 0.00 | 4141 | 0 | 0.00 | 0 | 0.00 | **4907** | 2 | 0.41 | 0 | 0.00 |  |
| Guge Boyre | 137 | 2 | 14.59 | 0 | 0.00 | 534 | 0 | 0.00 | 0 | 0.00 | 3626 | 0 | 0.00 | 0 | 0.00 | **4297** | 2 | 0.47 | 0 | 0.00 |  |
| Guge Shale | 208 | 0 | 0.00 | 0 | 0.00 | 812 | 4 | 4.93 | 0 | 0.00 | 5515 | 0 | 0.00 | 0 | 0.00 | **6535** | 4 | 0.61 | 0 | 0.00 |  |
| Guge Gaza | 130 | 0 | 0.00 | 0 | 0.00 | 505 | 0 | 0.00 | 0 | 0.00 | 3429 | 0 | 0.00 | 0 | 0.00 | **4063** | 0 | 0.00 | 0 | 0.00 |  |
| Zinge | 37 | 0 | 0.00 | 0 | 0.00 | 142 | 0 | 0.00 | 0 | 0.00 | 967 | 0 | 0.00 | 0 | 0.00 | **1146** | 0 | 0.00 | 0 | 0.00 |  |
| **Bobe Noyere Cluster** | **907** | **2** | 2.21 | **0** | 0.00 | **3530** | **4** | 1.13 | **0** | 0.00 | **23985** | **0** | 0.00 | **0** | #DIV/0! | **28422** | **6** | 0.21 | **0** | 0.00 |  |
| **Total** | **3436** | **436** | 126.89 | **3** | 0.69 | **13378** | **798** | 59.65 | **2** | 0.25 | **90901** | **606** | 6.67 | **1** | 0.17 | **107715** | **1840** | 17.08 | **6** | 0.33 |  |
